# The effect of cane vigour on the kiwifruit (*Actinidia chinensis*) and kiwiberry (*Actinidia arguta*) quality

**DOI:** 10.1038/s41598-021-92161-8

**Published:** 2021-06-17

**Authors:** Aljaz Medic, Metka Hudina, Robert Veberic

**Affiliations:** Department of Agronomy, Biotechnical Faculty, University of Ljubljana, Jamnikarjeva 101, 1000 Ljubljana, Slovenia

**Keywords:** Plant sciences, Secondary metabolism

## Abstract

Kiwifruit has not been studied as much as other well-known fruits especially when it comes to studies about plant vigour and training systems. The aim of the study was to determine the importance of cane vigour of *Actinidia chinensis var. deliciosa* ‘Hayward’ and *Actinidia arguta* ‘Issai’ in order to develop the proper pruning technique that results in the best fruit quality. In addition, the effect of storage parameters such as weight, firmness and quality of the fruit was also studied. The study showed that the fruit size and weight are lower in low vigour canes in *A. arguta*, in contrast to *A. chinensis*, where the fruit size and weight are smaller on high-vigorous canes. For *A. arguta*, it is recommended to choose high-vigour canes as the optimal fruit wood during pruning. In this way, the fruits will ripen more evenly. The other possibility is to perform the harvest two to three times per season to achieve a more uniform fruit quality. In the case of *A. chinensis* the fruit are less variable between different cane vigour, so harvesting can be done in a single picking. In *A. chinensis* the less vigorous canes tend to show a slightly better fruit quality.

## Introduction

Commercial production of kiwifruit started in New Zealand in 1930, and in 1960 the first kiwifruit orchards were established in Europe, especially in Italy and France. The *Actinidia* species comprise 54 species and 21 varieties, a total of about 75 taxa, of which only three are economically important for kiwifruit production: *A. chinensis*, *A. ar*guta and their hybrid cultivars^1^. Due to this short period of commercial production, not a lot is known about these fruit species^2^.

The most commonly grown cultivar is *A. chinensis* var. *deliciosa* ‘Hayward’. The fruits of *A. arguta* are quite different from those of *A. chinensis*, they are smaller, not covered with trichomes, so we can eat them whole without peeling the skin^1,3^.

For commercial production, the economy of fruit production is of crucial importance. Higher vigour canes produce higher number of fruits, because of the higher number of winter buds and consequently higher number of burst buds as well as higher carbohydrate availability^4^. Fruits are supposed to be larger on longer canes and closer to the base of the cane^5^.

Kiwifruit are generally harvested at the technological stage of fruit maturity, while fruit is still hard and not ready for consumption. The time of picking is usually determined by measurements of firmness, total soluble solids and dry matter^6^. During storage, parameters such as kiwifruit weight, firmness, sugars, organic acids and total phenols change, fruit weight, firmness, organic acids decrease, while sugars and total phenols increase^7^. Storage is very important as it affects the organoleptic properties and taste of the fruit. *A. chinensis* 'Hayward' can be stored for up to 6 months at a temperature between 0 and 1 °C^8^, while cultivars of *A. arguta* can be stored for up to 28 days^9^ at a temperature between 1 and 2 °C^10^.

Fruits containing more sugars are generally more sought after by consumers. Unripe fruits of *A. chinensis* contain less total and individual sugars than ripe fruits^3,11–14^. *A. chinensis* and *A. arguta* differ both in their total sugar content and in the proportion of individual sugars. Fruits of *A. chinensis* contain about equal shares of glucose and fructose as well as only up to 15 to 18% of sucrose^3,11,12^ while in fruits of *A. arguta* the share of sucrose can be up to 80%^3,15^. The difference in the sucrose content of fruits between the two species could be explained by the lower invertase activity in the last ripening stages and the higher sucrose phosphate synthase activity in fruits of *A. arguta* compared to *A. chinensis*^16^. Total sugars increase during ripening and after storage, but the ratio between the individual sugars remains the same^17^.

Besides sugars, organic acids are the most important compounds that affect the fruit taste. They give the fruit an indistinct flavour, as well as reduce bacterial infections that spoil the fruit^3^. The fruits of *A. chinensis* contain equal shares of citric acid and quinic acid and only up to 10% of malic acid^3,11,17^, while the fruits of *A. arguta* citric acid is predominant followed by quinic acid and malic acid^3^. Organic acids remain at the same level or decrease slightly after storage^17^.

Phenolic compounds are secondary metabolites, plants synthesise them during development in response to stress. The most common group of phenolic compounds are flavonoids, which are widely distributed in the plant and have many biochemical and pharmacological effects that have a positive impact on human health^18^. Phenolic content in the kiwifruit is highest in the protective peel, in *A. arguta* up to 15 times higher than in the pericarp. Since we eat fruits of *A. arguta* whole, without peeling, the amount consumed is much higher than in *A. chinensis* cultivars, up to three times higher in commercially important cultivars. A high phenolic content, combined with vitamin C, is usually associated with the antioxidant effect of the fruit^19^. Storage has a positive effect on the total phenolic content of the kiwifruit, due to the changes in phenolic metabolism and the higher activity of the PAL enzyme^20^. Therefore, the total phenolic content after storage usually increases from 5 to 10 mg per 100 g fresh weight after 4 months storage at 0 °C^13^.

The main focus of this study is to find out how different cane vigour of *A. chinensis* and *A. arguta* affects the fruit size and the fruit quality. Work in terms of determining the best canes for the highest fruit quality was carried out in order to re-evaluate the pruning techniques to confirm or modify the pruning guidelines for *A. chinensis* and *A. arguta*. Considering the previous work in the field, we expected lower vigour canes to produce smaller, lighter fruit of lower fruit quality than higher vigorous canes. It was expected that after storage, the total and individual sugar content in the fruits would be higher and the total and individual content of organic acids, total phenols and firmness would be lower. Only fruit of *A. chinensis* was measured after storage, as fruit commercially stores up to 6 months under the right conditions, while fruit of *A. arguta* does not store well and is usually put on the market immediately after harvesting.

## Materials and methods

### Plant material

The trial was carried out on *A. chinensis* ‘Hayward’ and *A. arguta* ‘Issai’ in 11-year-old kiwifruit orchard, located in Štatenberg, Makole, Slovenia (altitude 323 m; 46° 20′ 02′′ N; 15° 39′ 32′′ E) grown on a pergola trellis system, with a planting density of 3 × 4 m for both cultivars. The orchard has optimal growing conditions, with deep soil (8 m to bedrock), proper soil texture and yearly recommended fertigation based on the soil analysis. The yearly rainfall is between 1000 and 1100 mm and the average temperature was Jan (3.8 °C), Feb (− 0.6 °C), Mar (3.8 °C), Apr (15.1 °C), May (17.7 °C), Jun (20.2 °C), Jul (21.9 °C), Avg (22.2 °C), Sep (17.1 °C), Okt (12.5 °C), Nov (7.1 °C), Dec (1.8 °C). Cultivar ‘Issai’ is a hybrid between *A. arguta* and *A. rufa*. ‘Issai’ has been used as a representative of the *A. arguta* hybrids as it is a very fertile cultivar often commercially grown in many countries with a favourable climate, such as Japan, Portugal, Italy or Austria^21^. ‘Hayward’ was used as a representative of *A. chinensis* as it is the most widely grown cultivar worldwide. Natural pollination was used, the polinators were *A. chinensis* ‘Tomuri’ for ‘Hayward’ and *A. arguta* ‘Meader’ for ‘Issai’. Cultivar ‘Tomuri’ was in 1:5 ratio with ‘Hayward’ and ‘Meader’ 1:8 with ‘Issai’. All the analyses (fruit weight, size, firmness, total and individual sugars, total and individual organic acids and total phenolic compounds) were analysed in a laboratory of Biotechnical faculty, Department of Agronomy located in Ljubljana, Slovenia.

The trial was established after winter punning 2018. On 20th of February (BBCH 00) for each species, a total of 30 canes (10 canes per vine) from three average vines per cultivar were selected and pruned to a length of 40–50 cm, and 10 canes per cane vigour were studied. Each vine served as a repetition for further morphological and biochemical analysis. For each cane diameter was measured 1 cm from the base of the cane and the canes were divided into 3 categories (Table 1). Categories were based on largest to smallest cane diameter, with canes of *A. chinensis* ranging from 12 to 6 mm and those of *A. arguta*, which are smaller, ranging from 3 to 7.5 mm. On 10th of May (BBCH 55) the flowers were manually thinned, leaving only the main flowers, full blooming appeared on 25th of May (BBCH 65), further on manual thinning of fruit was done on 24th of June (BBCH 74) to set the fruit load on each individual cane uniformly. Although kiwiberry thinning is not practiced in commercial orchards because it is too labor intensive, it was done so that the experimental procedure would be comparable between the two species and the results could be better interpreted and compared. The manual thinning of the fruits was carried out in such a way that each annual shoot had 1 fruit on low vigour cane, 2 fruits on medium vigour canes and 3 fruit on high vigour canes, both on *A. chinensis* and *A. arguta*. The total number of fruits examined for their quality was 60 per species, proportionally from different type of cane vigour. Experimental research and field studies on plants, including the collection of plant material, complied with relevant institutional, national, and international guidelines and legislation.

**Table 1:** Distribution of canes by their vigour/diameter.

Species	Cane vigour	Cane diameter (mm)
*A. chinensis*	Low	6.00–7.99
	Medium	8.00–9.99
	High	10.00–11.99
*A. arguta*	Low	3.00–4.49
	Medium	4.50–5.99
	High	6.00–7.49

### Fruit size, weight and firmness measurements

The first fruit measurements (height, diameter) were taken on 24th June, immediately after thinning of the fruit, followed by measurements in 1 month interval (24th June, 23rd July, 22nd August, 22nd September).The weight of the fruits was determined on the same day as harvest, i.e. for *A. arguta* on 5th October (133 days after full bloom (DAFB)) and on 20th October (148 DAFB) for *A. chinensis*. Fruit firmness was measured at harvest with a digital hand penetrometer (T. R. Turoni, Forli, Italia) with 2.4 mm tip, as recommended by Huajia et al. (2016). The fruit firmness for *A. chinensis* was also measured after fruits were stored for 1 month at 5 °C (on 20th November).

### Sample preparation

The samples were prepared according to Mikulic-Petkovsek et al.^22^. The extract for sugars and acids was prepared from 5 g of fruit pericarp without the peel of *A. chinensis* and 5 g of whole fruit of *A. arguta*, as the fruits of *A. arguta* are eaten without peeling and the fruits of *A. chinensis* must be peeled before consumption. Plant material was cut into small pieces and 25 ml of double distilled water was added to the test tubes of each sample. Samples were placed on a shaker for 30 min on room temperature. After they were put in a centrifuge (Eppendorf Centrifuge 5810 R) for 5 min at 5000 rpm at 4 °C. Samples were filtered through a cellulose filter Chromafil A-20/25 produced by Macherey–Nagel (Düren, Germany), transferred to a vial and stored at − 20 °C until analysed by high-performance liquid chromatography (HPLC).

Extract for phenols was prepared according to Mikulic-Petkovsek et al.^23^ in test tubes from 5 g of fruit pericarp without peel of *A. chinensis* and 5 g of whole fruit of *A. arguta*, and 10 ml of methanol with 3% formic acid was added. Test tubes were places in a cooled ultrasonic bath for 60 min. After the sample was centrifuged for 8 min at 8000 rpm, supernatant was filtered through a polyamide filter Chromafil AO-20/25 produced by Macherey–Nagel (Düren, Germany), transferred to a vial and stored at − 20 °C until the start of the analysis.

### Analytical methods

Primary metabolites were analysed using a HPLC system connected to a RI plus detector (Finnigan Surveyor, Thermo, San Jose, USA) for determination of individual sugars and a diode array detector (Dionex UltiMate 3000, Thermo Scientific, Waltham, USA) for analysing individual organic acids.

Analytical conditions for primary metabolites were as described by Mikulic-Petkovsek et al.^15^. For analyses of sugars a Rezex RCM-monosaccharide Ca + 2% (300 mm × 7.8 mm) HPLC column produced by (Phenomenex, Califoria, USA) was used. The column was operated at 65 °C, mobile phase was double distilled water, and the flow rate was 0,6 ml/min. 20 µl of sample was injected and analysed for 30 min. Sugars were determined with the help of external standards for glucose, fructose and sucrose (Fluka Chemie GmBH, Buchs, Switzerland).

A HPLC column Rezex ROA-organic acid H + 8% (300 mm × 7,8 mm) produced by Phenomenex was used to determine the organic acid content according to Mikulic-Petkovsek et al.^15^. The column was operated at 65 °C, mobile phase was 4 mM sulfuric acid in double distilled water, UV-detector was set to 210 nm and the flow rate was 0.6 ml/min. 20 µl of sample was injected and analysed for 30 min. Organic acids were determined with the help of external standards for citric, quinic and malic acid (Fluka Chemie GmBH, Buchs, Switzerland).

Quantification was based on comparison of peak areas of samples with standard solutions. Content of individual sugars and organic acids were expressed in g/100 g of fresh weight (FW) of fruits.

UV/VIS spectrometer (Lambda Bio 20, Perkin Elmer, Waltham, USA) was used to determine the total phenolic content. Total phenolic content (TPC) of extracts was assessed by the Folin–Ciocalteau phenol reagent method (Singleton et al.^24^). For TPC a 10 ml test tube was used in which 7.9 ml of water was pipetted. After 100 µl of extract and 500 µl of Folin–Ciocalteau reagent was added. The extract was in (1:2 ratio (v/v) with MeOH). The samples were placed on a room temperature for 4 min, after 1.5 ml of sodium carbonate (20% w/v) and the remaining water was added so we had exactly 10 ml. The tube was closed, shaked and put in the oven on 40 °C for 30 min. A blind sample was prepared as well, which had 100 µl of methanol instead of the extract. The absorbance was measured at 765 nm, for each sample we had 3-repetitions. Total phenolic content of kiwifruit was expressed as gallic acid equivalents (GAE) in mg/100 g FW of fruit.

### Statistical analysis

Data was arranged in Microsoft Excel (Microsoft, Redmont, USA) and statistical analysed with R commander. For the determination of statistical differences between fruit, grown on canes with different vigour, one-way analysis of variance (ANOVA) was used, with Tukey’s tests. Statistical means at 95% confidence level were calculated to determine the significances of the differences (p < 0.05). The data are expressed as means ± standard error (SE).

## Results

### Fruit size, weight and firmness by cane vigour

The cane vigour didn’t affect the dynamics of fruit growth in either *A. arguta* or *A. chinensis*, as shown in Table 2. In the first measurements in *A. arguta*, the fruit was largest in high vigour canes and smallest in low vigour canes, the trend was maintained throughout the measurements and the difference was also statistically different. The same relationship was observed with fruit weight, as shown in Table 3, where differences between fruit weight and cane vigour were observed. In *A. chinensis* the medium vigour canes bore the larger fruit compared to the high vigour canes.

**Table 2:** Measured height and diameter (mean ± SE, in mm) of fruit in 1-month interval (shown in days after full blooming (DAFB)).

Parameter	Cane vigour	30 DAFB	59 DAFB	89 DAFB	120 DAFB
*A. arguta* fruit diameter	Low	16.73 ± 1.39 a	18.17 ± 1.34 a	19.32 ± 1.14 a	19.82 ± 1.29 a
	Medium	17.54 ± 1.24 a	19.01 ± 0.91 ab	20.40 ± 1.02 b	20.84 ± 0.89 b
	High	18.51 ± 0.95 b	19.38 ± 0.81 b	21.15 ± 0.80 c	21.63 ± 0.78 c
*A. arguta* fruit height	Low	21.53 ± 2.02 a	23.54 ± 2.18 a	24.72 ± 2.21 a	24.91 ± 2.14 a
	Medium	22.08 ± 2.66 a	25.03 ± 1.55 b	26.37 ± 1.51 b	27.05 ± 1.45 b
	High	24.20 ± 1.12 b	25.75 ± 1.04 b	26.50 ± 0.97 b	27.18 ± 0.85 b
*A. chinensis* fruit diameter	Low	31.31 ± 1.12 a	40.96 ± 1.25 a	42.77 ± 1.35 a	45.78 ± 2.74 ab
	Medium	32.41 ± 1.14 a	42.33 ± 1.29 a	44.46 ± 1.17 a	46.39 ± 1.63 b
	High	31.36 ± 2.37 a	41.54 ± 1.72 a	43.47 ± 1.57 a	44.91 ± 1.83 a
*A. chinensis* fruit height	Low	46.74 ± 2.30 a	56.64 ± 3.57 a	59.83 ± 4.22 a	63.23 ± 3.56 b
	Medium	46.91 ± 3.96 a	57.43 ± 3.61 a	60.22 ± 3.75 a	63.50 ± 3.28 b
	High	44.50 ± 4.86 a	56.13 ± 2.54 a	58.65 ± 2.13 a	60.14 ± 2.05 a

Mean values followed by the same letter within a marked column do not differ significantly.

**Table 3:** Average fruit weight of *A. arguta* and *A. chinensis* in g (mean ± SE) after picking.

Cane vigour	*A. arguta*	*A. chinensis*
Low	6.65 ± 1.66 a	91.86 ± 9.59 a
Medium	7.05 ± 1.09 a	90.48 ± 10.37 a
High	8.36 ± 1.41 b	85.99 ± 9.77 a

Mean values followed by the same letter within a column do not differ significantly.

The fruit of *A. arguta* was the heaviest in high and medium vigour canes, while the fruit weight of *A. chinensis* did not statistically differ (Table 3). The results were not statistically different, except between the fruit from high vigour canes and the fruit from low/medium vigour canes in *A. arguta*. In *A. arguta*, the fruit from high vigour canes was 15.7% heavier than the fruit from medium vigour canes and 20.5% heavier than the fruit from low vigour canes.

The cane vigour affected fruit firmness in *A. arguta*, as shown in Table 4. The fruits of medium and high vigour canes produced the firmest fruit and low vigour canes the softest. The firmness did not statistically differ between the fruit of medium and high vigour canes, even thought, fruit from high vigour canes was for 23% more firm than fruit from medium vigour canes. In *A. chinensis* cane vigour didn’t affect the fruit firmness, there was no statistical difference between canes of different vigour. In the storage period, the fruit firmness dropped substantially. Surprisingly, after storage firmness measurements revealed that the fruits of medium vigour canes remained firmer than the fruits of high vigour canes although the differences were not statistically significant.

**Table 4:** Average fruit firmness (mean ± SE, in N) of *A. arguta* and *A. chinensis*.

Cane vigour	*A. arguta* at harvest	*A. chinensis* at harvest	*A. chinensis* after 1 month of storage
Low	6.78 ± 6.23 a	40.36 ± 1.68 a	3.66 ± 0.81 a
Medium	15.87 ± 5.53 b	41.66 ± 2.23 a	4.06 ± 2.90 a
High	20.61 ± 8.74 b	41.98 ± 2.53 a	2.60 ± 1.64 a

Mean values followed by the same letter within a column do not differ significantly.

### Total and individual sugars

The main sugar in *A. arguta* was sucrose (63%), followed by fructose (23%) and glucose (14%). In *A. chinensis*, the main sugars after harvest were glucose (49%) and fructose (43%), followed by sucrose (8%). The sugar content of both the individual sugars and the total sugars increased after storage, but the ratio between the individual sugars remained quite similar, with the main sugars being glucose (46%) and fructose (47%) followed by sucrose (7%). The cane vigour affected total and individual sugar content in *A. arguta*, as shown in Table 5. The fruits of medium and high vigour canes produced the fruit with the lowest total as well as individual sugars, and fruit from low vigour canes the highest. Total sugars as well as individual did not statistically differ between the fruit of medium and high vigour canes, even thought, fruit from medium vigour canes had 9.4% more total sugars, 13.6% glucose, 5.6% fructose and more 10% sucrose than fruit from high vigour canes.

**Table 5:** Total and individual sugars (mean ± SE, in g/100 g fresh fruit) in fruit of *A. arguta* and *A. chinensis*.

Parameter	Cane vigour	*A. arguta* at harvest	*A. chinensis* at harvest	*A. chinensis* after 1 month of storage
Total sugars	Low	9.07 ± 0.11 b	2.21 ± 0.29 a	7.53 ± 1.11 a
	Medium	5.63 ± 0.22 a	2.22 ± 0.14 a	8.15 ± 0.03 a
	High	5.10 ± 0.51 a	2.06 ± 0.14 a	7.43 ± 0.21 a
Glucose	Low	1.26 ± 0.12 b	1.08 ± 0.14 a	3.52 ± 0.09 a
	Medium	0.81 ± 0.05 a	1.08 ± 0.04 a	3.75 ± 0.01 a
	High	0.70 ± 0.12 a	1.00 ± 0.06 a	3.37 ± 0.09 a
Fructose	Low	1.74 ± 0.14 b	0.96 ± 0.09 a	3.61 ± 0.38 a
	Medium	1.43 ± 0.04 a	0.92 ± 0.06 a	3.74 ± 0.04 a
	High	1.35 ± 0.09 a	0.88 ± 0.06 a	3.50 ± 0.12 a
Sucrose	Low	6.08 ± 0.20 b	0.17 ± 0.07 a	0.40 ± 0.29 a
	Medium	3.39 ± 0.14 a	0.21 ± 0.38 a	0.66 ± 0.02 a
	High	3.05 ± 0.31 a	0.17 ± 0.39 a	0.56 ± 0.05 a

Mean values followed by the same letter within a marked column do not differ significantly.

No clear influence of cane vigour on individual and total sugars was noticed in *A. chinensis*. High vigour canes produced fruit with a slightly lower sugar content, but the difference was minimal and not statistically significant. Storage for one month resulted in substantial increase in total and individual sugar. Surprisingly at after storage measurements, fruit from medium vigour canes seemed to contain more sugars, which were less variable than sugars in fruit from low, or high vigour canes, however this fact should be further evaluated.

### Total and individual organic acids

There were no statistical differences between total and individual organic acids among treatments in neither *A. arguta* nor *A. chinensis*, as shown in Table 6. Interestingly fruits from low/medium vigour canes of *A. arguta* had the highest organic acid values and opposite in *A. chinensis*, the highest content of organic acids was observed in fruits of high/medium vigour canes, in both before and after storage measurements. The total organic acids in the fruits decreased after storage, with individual acids: citric acid decreased slightly, quinic acid remained at about the same level and malic acid increased slightly.

**Table 6:** Total and individual organic acids (mean ± SE, in g/100 g fresh fruit) in fruit of *A. arguta* and *A. chinensis*.

Parameter	Cane vigour	*A. arguta* at harvest	*A. chinensis* at harvest	*A. chinensis* after 1 month of storage
Total organic acids	Low	1.79 ± 0.05 a	1.92 ± 0.01 a	1.87 ± 0.13 a
	Medium	1.78 ± 0.13 a	2.01 ± 0.02 a	1.92 ± 0.01 a
	High	1.71 ± 0.15 a	2.02 ± 0.07 a	1.92 ± 0.03 a
Citric acid	Low	0.98 ± 0.07 a	0.97 ± 0.04 a	0.85 ± 0.03 a
	Medium	0.98 ± 0.08 a	1.00 ± 0.03 a	0.89 ± 0.03 a
	High	0.97 ± 0.04 a	1.02 ± 0.04 a	0.92 ± 0.05 a
Quinic acid	Low	0.47 ± 0.04 a	0.75 ± 0.02 a	0.80 ± 0.10 a
	Medium	0.49 ± 0.02 a	0.82 ± 0.07 a	0.78 ± 0.03 a
	High	0.44 ± 0.05 a	0.79 ± 0.07 a	0.76 ± 0.03 a
Malic acid	Low	0.34 ± 0.04 a	0.19 ± 0.02 a	0.21 ± 0.03 a
	Medium	0.31 ± 0.03 a	0.19 ± 0.03 a	0.24 ± 0.02 a
	High	0.29 ± 0.03 a	0.21 ± 0.01 a	0.24 ± 0.01 a

Mean values followed by the same letter within a marked column do not differ significantly.

### Total phenols

Total phenols (Table 7) were expressed in milligrams of gallic acid equivalents per 100 g of fresh fruit. There were no statistical differences between phenols and cane vigour in either *A. arguta* or *A. chinensis*, but in both species a slight tendency could be observed that higher vigour canes produced fruit with higher phenolic content. Total phenols increased just slightly in fruit after storage.

**Table 7:** Total phenols (mean ± SE, in mg of gallic acid equivalents/100 g fresh fruit) in fruit of *A. arguta* and *A. chinensis*.

Parameter	Cane vigour	*A. arguta* at harvest	*A. chinensis* at harvest	*A. chinensis* after 1 month of storage
Total phenols	Low	59.2 ± 23.56 a	53.3 ± 1.61 a	53.9 ± 5.71 a
	Medium	63.2 ± 7.80 a	56.7 ± 1.80 a	59.0 ± 0.71 a
	High	68.1 ± 5.40 a	57.0 ± 2.06 a	59.2 ± 2.21 a

Mean values followed by the same letter within a column do not differ significantly.

## Discussion

### Fruit size, weight and firmness by cane vigour

Volz et al.^5^ and Thorp et al.^4^ reported that larger and heavier fruits were on the higher vigour canes and closer to the base of the cane. Our results for the fruits of *A. arguta* support these findings, whereas the fruits of *A. chinensis* do not show a clear picture, as there were no statistical differences in fruit weight between canes of different vigour, but only in fruit size. Heavier fruit in low vigour canes, but smaller in shape compared to the medium vigour canes could be a result of the imperfect fruit shape, as fruit was measured at the equatorial section at the minimum diameter of the fruit. The reason for two opposing results between the plants in the same genus could be of high importance in managing and pruning of the vines, as different techniques could apply in *A. chinensis* and *A. arguta*. Firmness in storage acceptable kiwifruits of *A. chinensis* ‘Hayward’ must be between 80 and 100 N^6^ and that of edible fruit between 4 and 10 N^25,26^. Firmness in storage acceptable kiwifruits of *A. arguta* must be between 20 and 35 N and that of the edible fruit between 1.5 and 3.5 N^9^. In terms of fruit firmness higher vigour canes produce firmer fruit in *A. arguta*, the same, but not statistically significant, was observed in *A. chinensis*. No studies are yet known with which we could compare the results.

### Total and individual sugars by cane vigour

*A. chinensis* and *A. arguta* differ not only in their total sugar content but also in their sugar composition. The proportion of glucose (49%) and fructose (43%) in *A. chinensis* is much higher than that of sucrose (8%). The results in fruits of *A. arguta* are similar to those of Nishiyama et al. (2008), who reported that the sucrose content in *A. arguta* is much higher than the glucose and fructose content. In our experiment, the sucrose content was 63%, followed by fructose with 23% and glucose with 14%. The reason for the high sucrose content in *A. arguta* is due to lower invertase activity and increased sucrose phosphate synthase activity in the last stages of fruit ripening^16^.

Jordan et al. (2000) reported that total sugars increase in storage as starch converts to glucose and fructose during the ripening of the kiwifruit, which is consistent with our results where sugars in *A. chinensis* increased after storage up to 3.6 times compared to the content at harvest. While the total sugar content increased, the ratio between individual stayed fairly the same which is in accordance with MacRae et al. (1989) and Amodio et al. (2007).

Fruits from *A. chinensis* compared between different vigour canes had a similar sugar content, both before and after storage, while fruits from *A. arguta* differ quite a lot by different cane vigour. Fruits from low vigour canes had a 61% higher total sugar content than fruits from medium and 88% higher content compared to fruit from high vigour canes. This combined with the data on fruit firmness (Table 4) suggests that fruits from lower vigour canes ripen earlier than fruits from higher vigour canes. Based on the results, the picking of fruit in *A. arguta* is recommended to be done in 2 to 3 terms, taking the cane vigour into consideration.

### Total and individual organic acids by cane vigour

In *A. chinensis*, the organic acid ratio was 50% citric acid, 40% quinic acid and 10% malic acid, which matches the results of Marsh et al. (2004) and are similar to Nishiyama et al. (2008) as well. Compared to *A. chinensis*, the fruits of *A. arguta* have more citric (55% of total acids) and malic acid (16%) and less quinic acid (27%). In *A. arguta* the organic acid ratio was citric acid, quinic acid and malic acid, while Nishiyama et al. (2008) reported slightly higher citric and quinic acid and lower malic acid concentrations.

Marsh et al. (2004) reported that after storage at 4 °C, citric acid decreases, malic acid content increases and quinic acid content remains the same, which is comparable to our results where after storage at 5 °C, citric acid decreases (for 12%), malic acid content increases (for 17%) and quinic acid content remains almost the same (> 1% decrease).

The cane vigour did not affect the total or individual organic acids in either *A. arguta* or *A. chinensis*.

### Total phenols by cane vigour

The total phenols in *A. arguta* were 11–19.5% higher than in the fruits of *A. chinensis*, which contradicts the results of Leontowicz et al. (2016), in which *A. arguta* contains 2–3 times more phenols than *A. chinensis*. The differences in phenolic content between both *Actinidia* species is probably due to the fact that in case of *A. arguta* phenolics were measure in pulp with the peel because the fruit is eaten whole, while fruit of *A. chinensis* was pealed as the fruit is eaten without the peel. Peel is the outher barrier of the fruit and normally contains more phenols compared to pulp^27^. The total phenols measured in *A. arguta* were at 59.2–68.1 mg GAE/100 g fresh weight, while Mikulic-Petkovsek et al.^28^ and Krupa et al.^9^ report higher values. Total phenols measured in *A. chinensis* were higher, as those reported by Tavarini et al. (2008). Smaller differences in total phenols between our results and the results in literature could be due to the growing conditions and different climate, as well as production technology. After one-month storage at 5 °C, concentrations of total phenols were 53.9–59.2 mg GAE/100 g fresh weight and higher than reported by Tavarini et al. (2008) and Amodio et al. (2007) after one-month storage at 0 °C. This could be due to different storage temperatures, and length of storage and higher phenols of kiwifruit at harvest.

The average phenolic content in fruits of *A. chinensis* increased by 4% during storage, which was in accordance to Tavarini et al. (2008) that suggested that total phenolic content in kiwifruit rose after storage. Martinez-Tellz and Laufuente^29^ suggest that storage at cold temperatures alters phenolic metabolism and increases phenylalanine ammonia lyase PAL activity. This is the crucial enzyme that initiates the phenolic synthesis.

Even if there was no statistical difference, higher vigour canes produced fruits with slightly more phenols in both *A. chinensis* and *A. arguta*, therefore cane vigour could affect the total phenolic content, however further detailed studies should be conducted.

## Conclusions

Interestingly, the fruits of *A. chinensis* from medium vigour canes showed the least variability in sugar, organic acid and phenolic content after storage, which suggests that medium vigour canes produce the most uniform fruit thus minimising losses due to repackaging and uneven ripening. It appears that cane vigour does not affect the fruit quality, with the exception of the fruit shape as previously reported by Volz et al. (2008), that demonstrated that higher vigour canes produce a higher proportion of misshaped fruit.

In *A. arguta*, the fruit showed characteristically uneven ripening. Data on total and individual sugars and fruit firmness indicate that the fruits of *A. arguta* ripen earlier from lower vigour canes. In order to achieve more uniform ripe fruits, harvesting should be done in 2 or 3 terms, taking cane vigour into consideration. With less variable fruit, storage could be better controlled and longer, as the fruit would ripen more evenly. With the right pruning techniques, better and longer market coverage could be achieved and losses due to repacking and uneven ripening would be minimised.

When comparing *A. arguta* and *A. chinensis*, a large difference was found mainly in the sugar content, with *A. arguta* containing eight times more sucrose than *A. chinensis*. The high sucrose content determined in *A. arguta* fruits could be explained 1st, by a lower invertase activity during the last ripening stages compared to *A. chinensis* or 2nd, by an increase in sucrosephos-phate synthase activity^11^.

The lack of research, particularly on the effect of cane vigour on kiwifruit quality and work on hardy kiwifruit (*A. arguta*), has been challenging, so pioneering work has been done in determining cane vigour and fruit load and its effect on fruit quality. The present study provides interesting data for further work on pruning, training systems and fruit quality for different *Actinidia*.
